# Stressors and Barriers to Services for Immigrant Fathers Raising Children with Developmental Disabilities

**DOI:** 10.1007/s11469-015-9584-8

**Published:** 2015-09-01

**Authors:** Nazilla Khanlou, Nida Mustafa, Luz Maria Vazquez, Nasim Haque, Karen Yoshida

**Affiliations:** Faculty of Health, York University, Toronto, ON Canada; Critical Care Services Ontario, Toronto, ON Canada; Department of Physical Therapy and Rehab Science Institute, University of Toronto, Toronto, ON Canada

**Keywords:** Canada, Developmental disabilities, Economic exclusion, Fathers, Gender roles, Immigration, Parenting, Social support

## Abstract

This narrative review examines research on the experiences of immigrant fathers raising children with developmental disabilities, and considers the findings within the Canadian context. Applying Green, Johnson & Adams’ (*Journal of Chiropractic Medicine*, 5(3), 101–117, 2006) methodology, a step-by-step process was followed to conduct the review. Four databases (PsychINFO, PubMed, CINAHL and Sociological Abstracts) were used for the search. A total of 39 articles were found to be relevant after applying the inclusion/exclusion criteria. Also 20 articles from published reference lists and peer-reviewed journal articles, located through Google Scholar, complimented the initial search. Along with House’s (1981) four dimensions of social support, an intersectional approach underpinned the analysis of findings. Four themes were identified which included: economic challenges, social influences, cultural influences, and the changing gender roles of fathers. Stressors and barriers to accessing health services in the post-migration setting were examined. Based on the review’s findings, the paper recommends 1) addressing income inequality, 2) improving access to health care, social and developmental services, 3) improving cultural-sensitivity of health care, social and developmental services, and 4) increasing participation of fathers. Overall, a more systemic understanding of immigrant fathers’ experiences is called for, taking into account their multiple social locations.

Parenting children with disabilities can embed intense and chronic caregiving responsibilities. The experience can be both a stressful and a rewarding dimension of parenthood. A number of factors influence the experiences of fathers, which can create a complex parenthood process depending on the disability of the child, as well as the availability of effective and efficient resources at the community level (Waldman et al. 2013). However, being a recent immigrant and falling into the category of racialized minority can further complicate the fathers’ experiences.

Research on the barriers faced by immigrant parents in terms of socioeconomic conditions, acculturation, and ability to access social programs and services for their child, remains scarce in the Canadian context (Daudji et al. 2011; Lai and Ishiyama 2004). Even more limited is information available on immigrant fathers raising children with disabilities, who often face unique stressors based on traditional gender roles and household responsibilities. This narrative review explores the experiences of immigrant fathers raising children with developmental disabilities.

Developmental disabilities (DDs) cover a range of disabilities which include intellectual disabilities, autism, Down’s syndrome, fragile X syndrome and other developmental delays. Children with DDs may have impairments and limitations affecting their physical-bodily function, their ability to perform daily activities, and their participation in routine behaviours such as going to school (Phillips 2012). In 2006, Statistics Canada reported roughly 3.7 % of children under the age of 15 having one or more DDs (Statistics Canada 2006). However, in Canada the percentage of children with disabilities growing up within immigrant families is not known (Khanlou et al. 2014).

Additional help in the form of social support is necessary for parents raising children with DDs (Gallagher et al. 2009; McConnell et al. 2014). However, 74 % of parents of the 3.7 % of children with disabilities living in Canada reported that extra help was too expensive for them, and many stated difficulty in finding and accessing appropriate resources (Statistics Canada 2006). For example, almost 40 % of those parents reported not knowing where to go to find help (Statistics Canada 2006). The situation for immigrant mothers and fathers of children with DDs is further complicated, as informal and formal social support systems are often diminished following migration. In 2011, 20.6 % of the total population in Canada was comprised of foreign-born individuals, making the country the highest population of immigrants among all G8 nations (Statistics Canada 2011). One in every five people in Canada is foreign born, and the numbers are rising yearly. For immigrant men specifically, unique challenges present themselves within the Canadian context; for example 7.5 % of recent immigrant men are not able to converse in English, and 36 % of immigrant men work part-time, not in full-time jobs (Chui 2011). These socio-economic factors may additionally cause further complexity for fathers raising children with DDs.

## Theoretical Approach

Parenting children with DDs involves persistent and long-term challenges with associated emotional impacts, such as stress for fathers and mothers who have limited social support. Social support is important in order to alleviate stress, anxiety, and frustration (Wang and Brown 2009). We apply House’s (1981) conceptualization of social support which entails four dimensions of social support: structural, instrumental, emotional and perceptive. As per House, *instrumental support* relates to financial help for families, as well as the availability of other forms of tangible/care giving services and programs. *Structural support* in the context of this review paper relates to the ease with which parents can access, utilize, and gain information about their child’s DD. Emotional and perceptive forms of support are more at the personal level in House’s model, with *emotional support* focusing on the social networks (for example, family and friends) present to help reduce caregiving demands; and *perceptive support* referring to the adequacy and helpfulness of support the individual feels they are receiving.

This paper adopts an intersectional approach to account for the multiple and complex processes which affect particular segments of the population, such as immigrant fathers of children with DDs in Canada. An intersectional lens allows for a simultaneous analysis of social difference and identity, which may link to “forms of systemic oppression…in ways that are complex and interdependent” (Dhamoon and Hankivsky 2011, p. 16). It is also a perspective that interrogates the power relations from which health disparities emerge (Jennings et al. 2014).

Jennings et al. (2014) argue that in order to consider immigrant families of children with DDs in contexts such as Canada, it is important to understand immigrant’s “complex social locations”, which are a result of various intertwined influences (p. 1648). They highlight the need to analyze such complexity beyond the sole description of social determinants of health, and through incorporating a more comprehensive analysis examining the “power dynamics” shaping immigrants’ experiences and disparities (Jennings et al. 2014, p. 1648). The objective of this review is to examine these very issues. The specific question the narrative review addresses is: “What does the literature tell us about the stressors impacting immigrant fathers of children with developmental disabilities, particularly in regards to social, cultural, and economic barriers?”

## Methods

A narrative review is a comprehensive narrative synthesis of previously published information (Green et al. 2006, p. 103). By following Green, Johnson and Adams’ (2006) method, and as described below, we applied a step-by-step procedure which includes identifying the sources of information used, the search terms, as well as the inclusion and exclusion criteria applied to narrow down the search.

### Sources of Information

Four electronic databases were used when completing this narrative review—PsychINFO, PubMed, CINAHL and Sociological Abstracts. All references found within these sources were organized and categorized using the online research management tool—RefWorks. The specific search within each of the four main databases was delimited through the inclusion and exclusion criteria.

### Search Terms and Delimiting

First, key search terms were used within each of the four data sources. The terms “father*” and “developmental disabilities” were used across all databases yielding 822 total search results. Originally the term “immigrants” had been added in order to limit results specific to immigrant fathers. However, this yielded very few results (i.e., PsychINFO, Pub Med and CINHAL presented only one article each), and therefore this term was removed. This further shows the limited literature in the area of immigrant fathers of children with DDs. Therefore the term “immigrants” was removed and the larger result of 822 articles across all four databases was delimited using the inclusion/exclusion criteria outlined below. Also initially, the term “Canada” was used to narrow the search in order to yield results within the Canadian context; however adding this term greatly reduced the number of articles as well. Therefore the term “Canada” was removed, which then yielded for a greater number of results. This further illustrates the limited research conducted on immigrant fathers’ within the Canadian context.

**Fig. 1:**
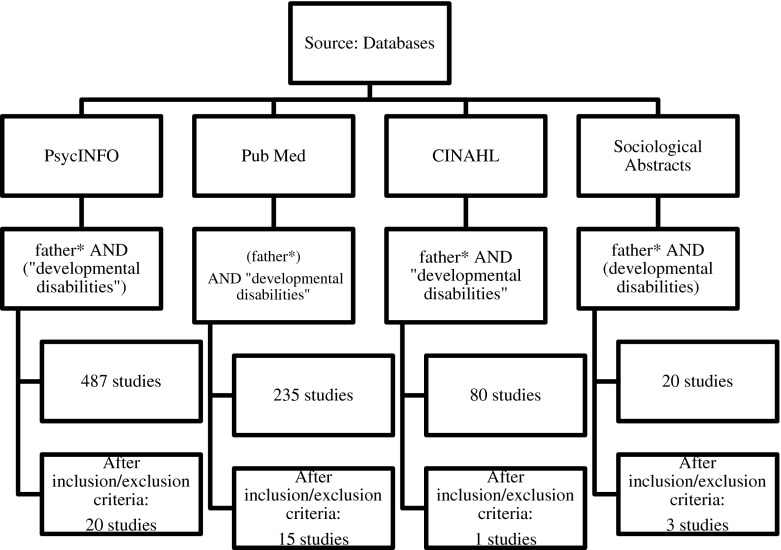
Literature review

Next, titles of each result were scanned within each database, according to the inclusion/exclusion criteria for the review. Lastly, paper abstracts were reviewed as a final step to ensure the included papers met the criteria set. Since we did not specify Canada as a key word, most of the literature reviewed was from the United States and United Kingdom. The years specified for the search were 1990 to 2015. Key words and final search results (before and after application of the inclusion/exclusion criteria) are shown in Fig. 1.

### Selection Criteria

The inclusion criteria included literature studying the experiences of fathers, the experiences of immigrant fathers, socioeconomic barriers of immigrant populations, and DDs in children (including impairment in body structure or function, inability to read or move around, and/or restriction in activity).

The selection criteria excluded literature that was not focused on fathers, literature that focused on disabilities caused by accidents (non-developmental disabilities), research exploring the experiences of fathers of adults as opposed to children, and non-English papers.

## Results

Using the inclusion/exclusion criteria, a total of 39 articles were found to be relevant across all four databases. The majority of articles were published outside of Canada, however they form part of the review to examine the various factors involved in fathering children with DDs. Findings have been applied within the Canadian context, as there is a lack of literature in this area.

The above search was complemented by additional search strategies. We used published references recommended by our peer scholar networks, as well as peer-reviewed journal articles located through Google Scholar. Key phrases used for this complimentary Google Scholar search were, “immigrant economic barriers Canada”, “immigrant employment Canada”, and “immigrant labor force Canada”. Through this step a total of 20 additional academic journal articles were included.

## Themes

The first part of the findings presents the challenges fathers face in general while parenting and caring for their children with DDs (“Raising Children with Developmental Disabilities” section). Along with these general challenges, immigrant fathers experience additional barriers that we present in the second section (“Immigrant Fathers: A “Double Burden”?” section) due to their immigration status in Canada.

### Raising Children with Developmental Disabilities

Fathers of children with disabilities experience differing levels of stress as they care for their family which is strongly linked with gender (Samadi et al. 2014; Oelofsen and Richardson 2006; Dyson 1997). Parents of children with disabilities living in Canada reported that their daily stress level varied from “a bit” to “extremely stressful” (Statistics Canada 2006). Added pressure on parents to balance personal and work life resulted in over 60 % of parents reporting “sometimes” to “always” feeling under stress. This strain affects all aspects of family life including parental marital satisfaction, parental physical and emotional health, as well as the ability to perform care giving tasks and responsibilities (Statistics Canada 2006).

The stressors that most commonly affect fathers of children with disabilities are both informational stress as well as practical stressors. Informational stress refers to the ongoing quest by fathers for medical information, in order to better understand the disabilities of their child. In one study it was found that not only do some fathers feel responsible for seeking information, but they also have pressure to balance family, work and routine daily tasks–leading to practical stress (Huang et al. 2012).

Some fathers have also expressed concern in regards to health care professionals viewing them as intrusive, assertive, and less competent than their wives (Huang et al. 2012). One reason given for the bias felt by men is the domination of women in the care giving field (e.g., hospital staff, nurses). Many fathers feel health care professionals are not supportive and depict fathers in a negative light (Pelchat et al. 2007). This causes further frustration and stress.

Fathers live more in the public domain, in which employment is a strong priority—to fulfill the “breadwinner” role (Darling et al. 2011; Gray 2003). In one study, it was found that some fathers show to be a strong power in the household, and a main authority in the family—in terms of financial support for the child in care (Huang et al. 2012). Taking on this role forces them to repress emotions, in order to be strong for the family and provide additional support for their wives. Huang, Chen and Tsai (2012) also found that some fathers tend to suppress the difficulties of adjusting to the new role as a father of a child with disabilities, as well as the difficulty of balancing work and family life—in order to be strong for the whole family.

A fear of loss of employment and financial support creates worry and guilt for fathers, related to the child’s medical care and treatment (Huang et al. 2012; Pelchat et al. 2003, 2007). Since most men in the household see their occupational role as primary, there is increased job stress in order to further support medical expenses (Darling et al. 2011; Brown and Barbarin 1996).

There are also structural limitations felt by fathers who want to be more involved in the life of their child with disabilities. Fathers feel a restriction by their employers, who are less flexible with their work schedule and give fewer accommodations in taking time off from work, compared with mothers. This leads to the stress of balancing family and work life, and at the same time fearing a threat of job loss (Darling et al. 2011).

Marital stress is experienced by both mothers and fathers of children with disabilities (Kersh et al. 2006). According to some researchers, the lack of intimacy, time, and communication, causes increased frustration between the spouses—who begin to view their relationship as a business (Goble 2004; Huang et al. 2012). As wives want to spend more time with their spouse, husbands tend to spend time away from home in order to strengthen their relationship (Darling et al. 2011). However, and conversely, having children with DDs can also lead couples to be more interdependent on each other, as they take turns in caregiving and providing temporary respite to each other.

The stigma felt by fathers in regards to their children’s disabilities, and the negative perception from outsiders, are among the stressors that fathers endure (Darling et al. 2011; Brown and Barbarin 1996; Shin et al. 2006). The specific characteristics of the child’s DD as well as worry for his/her future are also causes for great tension (Goble 2004; Pelchat et al. 2003, 2007; Gray 2003).

These stressors are reported by some researchers to be tied to decreased life satisfaction for fathers (Darling et al. 2011; Huang et al. 2014). Also among some fathers, increased depression due to the disabilities of the child may lead them to distance themselves or become stoic towards the child as a way of coping (Barak-Levy and Atzaba-Poria 2013). This social isolation (retreating from child, family, and friends), can harbor feelings of shame, sadness, anger and disappointment. The father can feel overwhelmed with a loss of control in his life, leading to poor physical health (Goble 2004; Huang et al. 2012). Avoidance and isolation have been linked with lower levels of wellbeing over time (Glidden and Natcher 2009; Glidden et al. 2006). In contrast, fathers who display emotional stability and agreeableness have decreased parenting stress (Vermaes et al. 2008).

There also is limited professional help offered for fathers living with a child with disabilities, and many fathers request more details of the DD (Boström and Broberg 2014). This lack of knowledge leads to feelings of discomfort, and can further cause a stressful environment (Darling et al. 2011). Table 1 summarizes these findings, as well as the number of relevant articles found within each thematic area.

**Table 1:** Sources of stressors on fathers

Sources of stress	Examples	Number of articles relevant
Financial	Fear of loss of employment	4
Informational	Responsible for seeking medical information	2
Social Isolation	Repressed emotions;	6
	Difficulty balancing work and family;	
	Increased depression;	
	Avoidance and isolation	
Workplace	Restriction by employers	1
Perceptions/Stigma	Health care professionals view fathers in a negative light;	9
	Little professional help available	
Marital	Lack of intimacy, time and communication	4

### Immigrant Fathers: a “Double Burden”?

Along with facing the stressors highlighted above, immigrant fathers experience additional stressors which can affect the way they deal with the DD of their child. We present below literature review findings on the contextual experiences of immigrant fathers who, we argue, carry a “double burden”. In addition to parenting and caring for their children with DDs, immigrant fathers experience unique stressors and barriers due to their immigrant status.

#### Socioeconomic Barriers

There are structural socioeconomic constraints that immigrants in Canada navigate on an everyday basis. Approximately 78 % of immigrants arriving to Canada within the last ten years are identified as a visible minority—individuals who are non-white in colour or ​who are non-Caucasian in race (Statistics Canada 2013). Being both a new immigrant and part of a visible minority group limits individuals and their families in terms of employment and income. In Canada, 1 % of recent immigrants live below the poverty line; this population has three times more probability to live in poverty than people born within the country (Affiliation of Multicultural Societies and Services Agencies of BC 2013). In addition, over a period of a decade after their arrival, the poverty rates remains high for this population, the rates are two times as high as non- immigrants (Ibid).

The literature points towards two socioeconomic characteristics of children with DDs in Canada: they are “more likely to live in poverty” than children without any disability, and they are “more often being raised in single-parent families”, particularly by single mothers (Jennings et al. 2014, p. 1650). The effects of living in families with lower income on young children include negative outcomes such as decreased educational attainment, and increased behavioral problems (Mayor 2010). These effects can be seen to carry on into adulthood, with children from low income families earning less as adults themselves (Mayor 2010).

Immigrant fathers of children with disabilities have increased stress through financial barriers and social isolation (Welterlin and LaRue 2007; Waldman and Perlman 2008). Goldring and Landolt (2012) argue that the “labor market incorporation of immigrants is important for social inclusion because it determines employment income and shapes material well-being” (p. 10). However, immigrants’ insertion into the labor market is one of the most “hardest tasks” (Statistics Canada 2005, p. 72). Scholars have highlighted the difficulties immigrants encounter in their job search as well as the lower earnings they receive compared to the non- immigrant population (Frank 2013; Walters et al. 2006; Teelucksingh and Galabuzi 2005). Precarious, unstable, and low pay jobs have negative impacts on immigrant families’ psychological, material, and physical health and well-being (Zeynep and Berry 1996). Fathers’ unstable employment may also have a direct impact on their ability to provide care to their children with DDs. For example, in precarious work environments, it is unlikely that fathers will get support from their supervisors to skip job hours in order to attend their children’s health, education, or developmental services appointments.

Economic and material constraints affect how parents manage and provide care to their children with DDs. For example, basic practical tasks such as attending medical visits and doctor appointments may become an obstacle due to transportation limitations, or lack of a valid driver’s license for fathers (Welterlin and LaRue 2007). This is of special relevance as new immigrant families may settle in peripheral areas of cities (where rent is cheaper), while services are mainly located centrally.

The intersection of migration status and access to services is determined by variables such as gender, age, class and ethnic background (Oxman-Martinez and Hanley 2011). Along with their economic exclusion or instability, studies have shown that immigrant legal status determines the quality of and access to public services. Villegas (2013) argues that migrants with precarious legal status “have insecure access to a range of goods and services that in Canada are considered essential and linked to the basic rights and entitlements of citizenship” (p. 222). Even though health and developmental services for children with DDs exist, contingent factors such as fathers’ legal status determine their right to access and use services. Therefore, as Jennings et al. (2014) argue, “immigrant status is itself a social determinant of health” (p. 1647).

#### Social Isolation

Along with the above material and economic constraints, the literature points towards new immigrant families experiencing social barriers. For example, immigrant families leave strong social networks (i.e., close family and friends) in their home countries, and therefore can feel isolated and alone in navigating through a new environment of services. Jennings et al. (2014) explain that “society stigmatizes disabled children and views disability in childhood as a social problem” (p. 1649); and parents feel this strong stigma of their child’s DD by their own support systems—who tend to blame the parents for the child’s condition (Daudji et al. 2011; Kramer-Roy 2012). Therefore, immigrant families may be hesitant to readily seek out support in their new country of resettlement.

#### Language and Cultural Barriers

Other important intersecting factors affect not only access to services but also act as barriers to immigrant fathers’ full enjoyment of the benefits of such services. Discrimination at the structural level is quite often felt by immigrant fathers who have children with disabilities (Lindsay et al. 2012). Experience with health care professionals has been highlighted as a barrier to care for immigrant parents, who may view providers as superficial and uncaring (Kramer-Roy 2012). They can feel alienated by the health care system, mainly due to communication barriers and cultural differences (Fellin et al. 2013; Ali et al. 2001; Waldman and Perlman 2008).

The language barrier is one of the main obstacles in accessing, receiving, and utilizing health care services for parents of children who have disabilities (Lindsay et al. 2012). For many immigrant fathers, English is not their first language; in addition medical language is in itself complex, potentially leading to deficient communication between fathers and health care professionals. This may impact on the ways fathers understand the condition and diagnosis of their children; and may also prevent them from accessing and using necessary services for their children (Ali et al. 2001; Lai and Ishiyama 2004; Waldman and Perlman 2008; Fellin et al. 2013; Kramer-Roy 2012; Welterlin and LaRue 2007). Despite the use of translators, families who have limited proficiency in English may still feel uncomfortable and frustrated trying to obtain information about their child’s DD.

There is potential for miscommunication by health care professionals due to differing concepts of medical diagnosis and treatment. Immigrant fathers can often have difficulties in understanding the “Western” definition of their child’s condition, the medical reasons behind it, and the treatment regime necessary (Lindsay et al. 2012). According to Greeson et al. (2001) there is a subtle fear felt by immigrant parents towards ‘Western medicine’—as these beliefs may conflict with non-Western ideals. For example, a child’s disability in one specific culture may be viewed as a punishment from God rather than an entirely medical condition—a more spiritual perspective rather than medical (Welterlin and LaRue 2007; Kramer-Roy 2012). Therefore, some immigrant fathers may be unwilling to accept the Western medical diagnosis of a condition, and may see their child as mis-labelled (Daudji et al. 2011). This discrepancy can lead to a lack of knowledge about the child’s condition and about the health care system including available programs and services (Ali et al. 2001; Welterlin and LaRue 2007; Fellin et al. 2013).

Finally, another important factor is the process of acculturation for some first generation immigrant families. Families who migrate to a new country tend to preserve their unique cultural values, norms and traditions (Ali et al. 2001; Lai and Ishiyama 2004). The process of migration to a new host country comes with challenges in terms of adaptation to a set of differing beliefs, ideologies, and practices. Immigrant fathers may experience increased stress through this process, having difficulty with relocating and adjusting to a new environment (Lai and Ishiyama 2004). The pressures of adapting to a new culture, along with taking care of children with DDs, increase the burden of care for these parents.

#### Gender Roles

Gender roles tend to shift for mothers and fathers after migration. Mothers become involved within the family in new ways through working, earning money, and playing a vital role in hospital visits and appointments. This change in roles can bring stigma to fathers, who are viewed within their own communities as not being strong and dominant in their family unit—by not subscribing to traditional gender norms of being the family breadwinner (Wolff et al. 2010). Balancing these external stressors can make immigrant fathers even more vulnerable, as compared to non-immigrant fathers. Table 2 summarizes the findings of this section, as well as the number of relevant articles found within each thematic area.

**Table 2:** Unique stressors and barriers for immigrant fathers

Source of stressor	Examples	Number of articles relevant
Financial	Lack of adequate employment and income; Poverty	11
Social Isolation	Leave strong social networks in home countries	3
Perceptions/Stigma	Stigma related to both, gender roles and children’s disabilities; Negative view from family	4
	Parents to blame for child’s condition	
	Health care providers lack of understanding	
Practical Barriers	Transportation limitations	1
Gender	Shift and reversal of gender roles	1
Language	Miscommunication	8
Culture	Cultural values, norms and traditions; Fears towards Western medicine	8
Discrimination	Immigrant minorities social and economic exclusion	1
Legal Status	Lack or limited access to health care services	2

## Discussion

Findings of this narrative review indicate that immigrant fathers of children with DDs in Canada are impacted across all House’s (1981) four dimensions of social support. Immigration affects the availability of *instrumental support* for families due to under-employment and job insecurity following migration. Prevailing policies in the host country regarding access to services intersect with language barriers, as well as with unfamiliarity of available services, resulting in diminished *structural support*. Gender strongly impacts available *emotional support* for fathers in general and immigrant fathers in particular, as fathers’ social networks are dually reduced due to their children’s DDs and post-migration disruptions in family and friendship networks. Fathers of children with DDs in general may *perceive* services are biased towards favoring mothers (however, there is limited literature to ascertain whether immigrant fathers of children with DDs also feel this way in the initial phases of their resettlement in contrast to long-term immigrants).

Studies have associated the immigration process with health decline for immigrants after they arrive to Canada (Fuller-Thomson et al. 2011). Raising children with disabilities can also negatively impact parents’ health. This review supports the notion that while raising children with DDs can be emotionally and physically challenging, the task becomes more overwhelming when there is a lack of social support, such as is the case for new immigrant families. As Jennings et al. (2014) assert the main issue causing difficulty is not mothering (and in this review’s case fathering) children with disabilities, but rather the lack of support for the parent caregiver. New immigrants may not be aware of their basic rights to health and education, and therefore may not necessarily seek out these resources for their child (Khanlou et al. 2014).

## Recommendations

The following recommendations are proposed in relation to: a) income inequality and unemployment; b) access to health care, social and developmental services; c) cultural-sensitivity of health care, social and developmental services; and d) participation of fathers.

### Reduce Income Inequality and Unemployment/Underemployment

The increasing wage gap and employment differential between immigrants and native-born populations are among the important policy issues highlighted in the literature. Teelucksingh and Galabuzi (2005) show that in Canada, during the period 1996–2001, racialized groups and newcomers sustained “a double digit income gap and a higher rate of unemployment” than native-born workers (p. 3). Reitz (2007) also confirms that since the 1970s and until late 1990s immigrants’ employment rates and earnings have declined. These groups are also overrepresented in precarious low paying occupations (Teelucksingh and Galabuzi 2005). As Walters et al. (2006) explain the earning and employment gaps are important for immigrant populations in Canada and around the world, which has implications for entire populations.

Promoting the insertion of immigrant workers in stable, well paid and full time jobs will benefit immigrant families and the economy of the receptor country (Friedberg and Hunt 1995). Immigrants “represent important economic, social and demographic assets to their host country” (Asanin and Wilson 2008, p. 1271). The exclusion of this population from the economy has negative impacts on both immigrants and the host country since the latter is not being benefitted from immigrants’ productivity and professional skills (Teelucksingh and Galabuzi 2005). Immigrant’s employment may also impose a burden for the host economy “for dealing with poverty and its impacts on health and social well-being” (Teelucksingh and Galabuzi 2005, p. 3).

### Improve Access to Health Care, Social and Developmental Services

Disparities in access to health care services are determined by gender, age, race, income, language, and immigrant status—among others (Stewart et al. 2006). The lack of equitable access to health care, social and developmental services prevents disadvantaged groups to fully enjoy, participate and exercise their rights as citizens. Scholars highlight the need to reciprocate the benefits that immigrants bring to the host society in terms of accessing to their entitled social benefits (Asanin and Wilson 2008). Among the barriers identified to access health care services are language, cost, location, transportation and culture.

Policies in the host country shape the trajectories of inclusion and exclusion of its immigrant population (Stewart et al. 2006). Therefore to reduce barriers to accessing health care, social and developmental services for immigrant families raising children with DDs, as funders of these services, government policies need to promote funding of inclusive practice strategies, and equitable organization of delivery of services.

### Improve Cultural-Sensitivity of Health Care, Social and Developmental Services

Health care, social and developmental services professionals need focused education and training on providing gender-appropriate, culturally sensitive care for immigrant parents of children with DDs. Targeted education should be given on how to interact with groups who have language barriers, and help to empower parents to navigate through the multiple systems of services. Despite language and knowledge barriers, it is still important for service providers to build rapport and trust with immigrant fathers in support of the optimal development of their children with DDs. For example, health care providers should educate themselves on the economic and language barriers of newcomer immigrant families, and gendered perceptions felt by fathers, and take extra time to make parents feel comfortable. This will create a better relationship, impact the way parents perceive and interact with the health care system, and ultimately benefit the health of the child (Lindsay et al. 2012).

Family-focused interventions have been suggested to benefit immigrant communities, because of their focus on family behaviors and parenting styles (McCubbin et al. 1993). It may therefore be beneficial to engage in a whole family-approach when providing care to these communities. Research has also shown that home visits to families who have children with disabilities may be better for them, rather than the hospital setting (Fellin et al. 2013). Immigrant families can feel more comfortable at home, and may not be as intimidated by medical professionals within their own home environments.

### Increase Participation of Fathers

Fathers who challenge traditional gender roles tend to be more involved in their child’s life. Fathers who are actively involved with their child, and who provide positive care and support, have shown to benefit from the father-child relationship. Taking on nurturing roles for the child, in a playful manner, creates a stronger bond, and increases positive family coping (Simmerman et al. 2001). Fathers who take on this “playmate role”—as provider, protector, and observer—have better outcomes in terms of emotional satisfaction, in addition to better outcomes for their child with DD, and for the entire family (Huang et al. 2012). It has been recommended for fathers to work as a team with their spouses, rather than in a dominant manner, to provide for their child. By breaking these gender norms, husbands and wives increase support for one another, resulting in increased well-being (Wolff et al. 2010).

Social programs and services available solely for fathers are rare. Fathers report being ignored during family therapy activities, having their wishes ignored, and feeling excluded from the diagnostic processes for their child (Huang et al. 2012). Fathers can also feel anger towards receiving second-hand medical information, as some health care professionals choose to disclose information to mothers, without the presence of the father (Pelchat et al. 2007). This lack of education leaves fathers feeling uncomfortable, and causes them to distance away (Goble 2004).

Tailored counseling and support programs should be initiated for fathers only, focusing on stress management techniques, self-esteem, education, resources for coping, and emotional expression (Thompson et al. 2013; Trute 1995). Such approaches have shown to increase father involvement in the care of the child, as well as empowering them to feel significant in the family unit (Darling et al. 2011; Willoughby and Glidden 1995; Laxman et al. 2015). Individualized counseling approaches for immigrant fathers may also be necessary, recognizing the particular structural and instrumental social support barriers this population is facing (beyond the personal realm).

## Limitations of the Review

The findings and recommendations of this review are limited in light of a number of caveats: 1) The review integrated different types of DDs without distinguishing between them as well as their level of severity and associated caregiving intensity for fathers; 2) A specific search for the category of “single fathers” was not conducted, which may have provided us with the particular barriers faced in this context of parenting and caregiving of children with DDs; 3) Immigrants were examined as one category (due to the limited literature available on refugee fathers of children with DDs); however, we recognize that migrants are not a homogenous group; and 4) Immigrant families’ specific pathways to Canada and their prior home country experiences may differently shape their experiences with the Canadian systems of support for families of children with DDs. It was not possible to individualize the review findings to this level of specificity due to a lack of sufficient numbers of ethnoculturally specific studies on immigrant parents of children with DDs.

## Conclusion

Gender roles, divisions of labor, and parental responsibilities create unique experiences for both mothers and fathers. Most research has focused on the role of mothers as caretakers of children with disabilities; and fathers have often been unobserved in this realm (Johnson and Simpson 2013). By conducting a narrative review of the literature, we provide an analysis of the stressors and barriers experienced by fathers of children with DDs in general and immigrant fathers in particular. Immigrant fathers experience additional stressors and barriers compared to their non-immigrant peers due to post-migration related disruptions in social support. Future research is needed that unpacks broad categories of “immigrant” identity and DDs so to translate findings into specific policies and practices. Also while most of the research to date has focused on the challenges of parents raising children with DDs, day-to-day narratives of service providers and families point toward the tremendous resilience of parents in the context of raising children with DDs. Future research is called for that documents the families’ agency in light of ongoing parenting-caregiving demands, despite limitations of available social support.
